# Patient decision aids for patients with differentiated thyroid carcinoma: development process and alpha and beta testing

**DOI:** 10.3389/fendo.2023.1162537

**Published:** 2023-05-31

**Authors:** Anna Koot, Rosella Hermens, Petronella Ottevanger, Romana Netea-Maier, Peep Stalmeier, the COMBO study group, Marieke Snel

**Affiliations:** ^1^Radboud Institute for Health Sciences, Department for Health Evidence, Radboud University Medical Center, Nijmegen, Netherlands; ^2^Department of Internal Medicine, Division of Endocrinology, Radboud University Medical Center, Nijmegen, Netherlands; ^3^Radboud Institute for Health Sciences, Scientific Institute for Quality of Healthcare (IQ Healthcare), Radboud University Medical Center, Nijmegen, Netherlands; ^4^Department of Internal Medicine, Division of Oncology, Radboud University Medical Center, Nijmegen, Netherlands

**Keywords:** thyroid cancer, shared decision making, surgery, tyrosine kinase inhibitor, decision aid

## Abstract

**Background:**

Patient decision aids (PtDAs) are structured clinical tools that facilitate shared decision-making. Two important treatment decisions for patients with differentiated thyroid cancer (DTC), which could benefit from PtDAs, are as follows (1): the extent of surgery decision in patients with low-risk DTC and (2) the decision to start or delay starting the treatment with tyrosine kinase inhibitors (TKIs) in patients with advanced tumors.

**Material and methods:**

PtDAs for these two decisions were developed using the International Patient Decision Aids Standards (IPDAS) quality criteria in an iterative process of prototype development *via* alpha and beta testing by patients and physicians. The information content of the PtDAs was based on the available literature, current guidelines, and patient’s needs, preferences, and values.

**Results:**

The web-based PtDAs underwent two rounds of alpha testing, revisions, and beta testing. The PtDAs have the same structure, consisting of six steps: a general introduction, information about the treatment options, comparing the treatment options, knowledge questions, a values clarification exercise, and saving the information. The alpha testing (*n* = 8 patients, *n* = 10 physicians) showed that the PtDAs were highly acceptable and usable for decision-making. Results of the beta testing in 20 patients showed that two patients did not use the PtDA; the other 18 patients found that the PtDAs were readable (*n* = 17) and helpful (*n* = 14) for decision-making. All patients recommend using the PtDAs.

**Conclusions:**

Evidence-based PtDAs were created for patients with DTC for two different treatment decisions. Our final version was judged to be clear, balanced, and helpful in decision-making.

## Introduction

Differentiated thyroid cancer (DTC) is rapidly increasing in incidence throughout the world, mostly as a result of the increased use of diagnostic imaging and surveillance (1–3). Primary treatment for most patients with DTC consists of surgical removal of the thyroid (total thyroidectomy), followed by treatment with radioactive iodide (I^131^, RAI) to ablate the remaining thyroid remnants or destroy (microscopic) DTC remnants. This is followed by life-long thyroid hormone therapy (4, 5). In patients with low-risk tumors smaller than 1 cm, the removal of only the affected lobe (lobectomy) is currently the standard of care. These patients do not require RAI treatment, and most of them maintain normal thyroid function. Several studies, including a recent meta-analysis (6), suggest that this more conservative approach results in similar long-term outcomes and therefore could be applied also in selected patients with low-risk tumors larger than 1 cm. However, because there are no prospective randomized controlled clinical trials (RCTs), comparing different surgical approaches for patients with low-risk DTC larger than 1 cm, management recommendations are currently based on retrospective data (7). After primary treatment, the majority of patients with DTC have an excellent long-term prognosis (1, 8–11).

Nonetheless, after primary treatment, up to 30% of the DTC patients develop recurrent disease and/or distant metastases. These patients have a less favorable prognosis amounting to an average life expectancy of 3–5 years for those with tumors that are nonresponsive to RAI (12, 13). For patients with metastatic disease, both local and systemic treatments are available, but overall complete remission is only seen in one-third of cases (14) and, while these treatments can improve DTC-related symptoms and disease-free survival, there is no evidence that these treatments result in a clear improvement of the overall survival (OS).

Therefore, for both patients with low-risk DTC and with RAI- refractory advanced DTC, there is a discussion about the optimal treatment strategy that better balances the risks and benefits for the individual patients and their personal preferences. As such, some patients with low-risk DTC might currently undergo overtreatment, which could negatively affect their quality of life (QOL) (6).

The current guidelines of the American Thyroid Association (ATA) state that less aggressive therapy, for example, a thyroid lobectomy and a tailored follow-up, can be equally acceptable and explicitly mention room for patients’ preferences (15). Similarly, for asymptomatic or mildly symptomatic RAI- refractory DTC patients, an important unanswered question regards the optimal timing of starting tyrosine kinase inhibitors (TKIs). For those who are asymptomatic or only mildly symptomatic, starting the treatment too early may expose them to side effects and worsen their QOL without evidence of a survival benefit (16). The recent European Thyroid Association (ETA) guidelines state that the decision to start TKIs should include patient-related medical factors and patients’ preferences with respect to treatment goals and patient values, and acceptance of adverse effects (17).

Given the different benefits and harms of surgery and TKI treatment, the care for patients with DTC should be better personalized. Patients could be informed using plain-language, evidence-based information about these decisions. Patient decision aids (PtDAs) are suitable instruments to support decision-making. Studies have shown that PtDAs generally improve patient knowledge, result in more realistic patient treatment expectations, increase active patient participation in decision-making, and reduce indecisiveness (4, 18). Currently, no PtDAs for DTC patients for the above-mentioned treatment decisions are available. This study presents the development process and alpha and beta testing of different PtDAs in order to provide decision support for two treatment decisions in patients with DTC (1): the extent of surgery in patients with low-risk DTC larger than 1 cm, and (2) the decision to wait or start with TKIs in patients with asymptomatic or mildly symptomatic advanced, RAI-refractory DTC.

## Materials and methods

### Development process

The development of the PtDAs was part of the Communication Booster (COMBO) study (NCT03905369), which aimed to develop, evaluate, and implement decision-support tools for DTC patients. Thirteen hospitals (six academic and seven non-academic) in the Netherlands participated, as well as the Dutch patient association “Schildklier Organisatie Nederland (SON).” In the study, we randomized patients into an intervention or control group. Patients in both groups received general information about DTC from their physicians. In addition, the intervention group received also the PtDA. The Medical Ethical Committee (CMO) of the region Arnhem–Nijmegen approved the study protocol (MEC-2018-4521).

**Figure 1:**
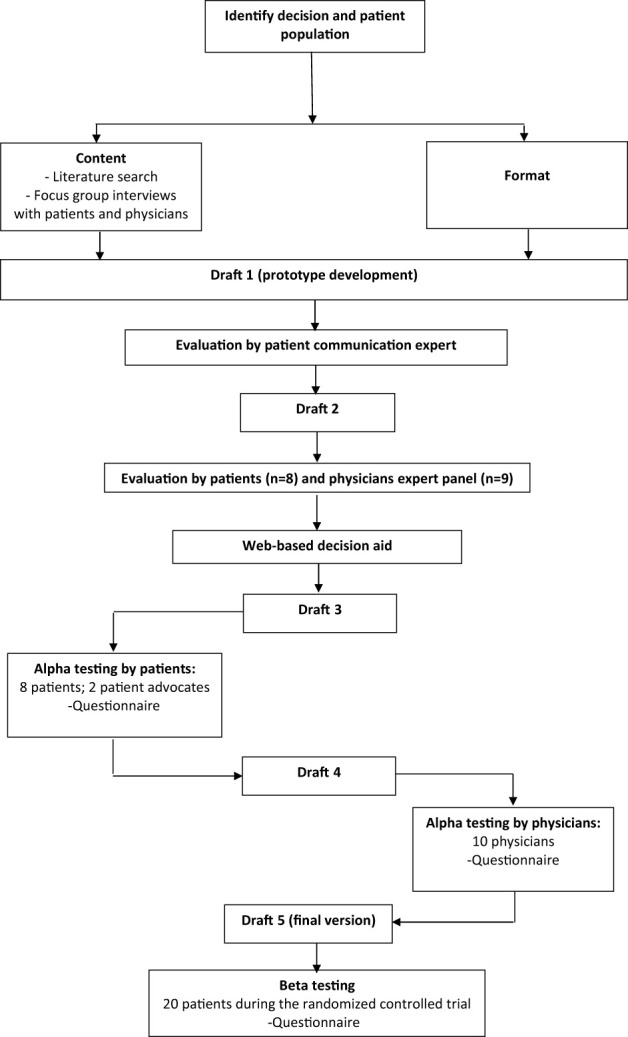
Development process of the patient’s decision aids.

The International Patient Decision Aids Standards (IPDAS) were used to guide the development of the PtDAs and were based on behavioral and decision-making theories underlying the Ottawa Decision Support Framework (19–21). To guide the development process, recommendations by Coulter et al. (21) and the Dutch Guideline were consulted (22). The development process is shown in **Figure 1** and was performed by a project group consisting of a PhD student who is also a medical doctor (AK), a decision-making scientist (PS), an endocrinologist (RN-M), an implementation scientist (RH), and an oncologist (PO), assisted by a patient and physician expert panel. None of these patients or physicians had any conflicts of interest.

#### Scope and purpose of PtDAs

**Figure 2:**
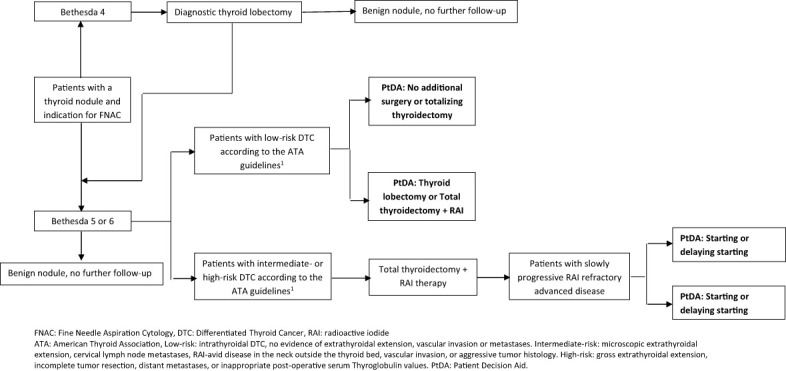
Overview of the different decision aids in the Netherlands.

The scope and purpose of the PtDAs were defined by the project group. Meetings were held with the project group to reach a consensus on the scope, purpose, and target audience. The main scope was to improve informed choice regarding two different treatment decisions in patients with DTC. Therefore, the project group agreed to develop a PtDA for each treatment decision (**Figure 2**). The target audience for the first PtDA was defined as patients with low-risk DTC (> 1 cm) according to the ATA criteria (15), considering the extent of thyroid surgery. For the second PtDA, the target group consisted of patients with advanced RAI- refractory asymptomatic or mildly symptomatic DTC considering whether to wait or start with TKI treatment.

#### Content and format

The first part of the COMBO study consisted of a literature review for clinical parameters, resulting in a meta-analysis of the extent of surgery decision (6), followed by focus groups with patients with DTC and physicians (endocrinologists, surgeons, oncologists, nuclear medicine physicians) treating patients with DTC to explore patients’ needs, preferences, and values regarding the two treatment decisions, which resulted in a focus group paper (23). Based on the identified needs, guidelines, literature review, and the expertise in the project group, domains were determined that were important for decision-making in this setting: (1) risks and (2) benefits of both treatment decisions; (3) oncological outcomes in both treatment decisions; and (4) patients’ personal values in decision-making. For the first three domains, we collected available literature (6, 24–26); for the fourth domain, values mentioned by patients and physicians were extracted from the focus group interviews (23). The development was an iterative process (**Figure 1**), with the content and format informed by the IPDAS guidelines. An online, web-based format was chosen by the project group to provide a PtDA that is tailored to a treatment decision. This resulted in the prototype draft.

### Methods of alpha & beta testing and revision

The first drafts of the two PtDAs (“thyroid lobectomy or total thyroidectomy” and “to wait or start with TKIs”) were finished after an iterative process of revising and reviewing the prototype content with the project group. In order to ensure the PtDAs were accessible to a broad audience, these drafts were evaluated on readability by communication experts experienced in increasing readability (“Stichting Makkelijk Lezen”) (27). The reading level aimed to enable 95% of the Dutch population to understand the text, level A2-B1, according to the Common European Framework of for Languages (28). This resulted in the second draft (29) (**Figure 1**).

The second draft was sent by email to two different panels, consisting of eight patients and nine physicians (**Figure 1**). The eight patients made a decision regarding DTC in the past (four with low-risk DTC and four with advanced disease). The nine physicians consisted of two endocrinologists, two surgeons, and five oncologists treating patients with DTC. Patients and physicians were asked to evaluate this draft of the PtDA on clear language, content, layout, and comprehensibility. They were recruited from participating Dutch academic hospitals. Feedback on the PtDAs was also given by two patient advocates recruited from the patient association SON (**Figure 1**). This resulted in a third draft with a web-based format.

This third web-based draft was developed and alpha-tested by patients who made a decision regarding DTC in the past (**Figure 1**). The patients were recruited by endocrinologists from the participating Dutch academic hospitals, who asked one or two of their patients to participate in a face-to-face interview about the PtDA. After verbal consent, patients were approached by the investigator (AK) by telephone. Eight patients (four with low-risk DTC and four with advanced disease) were willing to participate (**Table 1**). All interviews were conducted by AK, and further interviews were determined by data saturation. The first part of the interview was unstructured using a think-aloud method (30). The second part was semi-structured, and participants were asked to fill out a questionnaire on content, structure, length, readability, balance, comprehensibility, relevance, reliability, completeness, and usability (**Figure 1**; **Table 2**). This resulted in the fourth draft.

**Table 1:** Demographic characteristics of patients and physicians who participated in the alpha testing.

	Patients (*n* = 8)	Physicians (*n* = 10)
**Mean age in years (range)**	42 (35–72)	45 (36–62)
**Sex (*N*, %female)**	6	8
**Caucasian (*N*, %)**	8	10
Level of education		
High school or less	2	
Vocational education	4	
University	2	
Type of treatment		
Surgery		
Thyroid lobectomy	2	
Total thyroidectomy	2	
Systemic therapy		
Lenvatinib	1	
Sorafenib	3	
Type of professional (*N*, %)		
Endocrinologist		2
Surgeon		6
Oncologist		2
Type of hospital (*N*, %)		
Academic		4
Non-academic		6
Years of experience as professional (*N*, %)		
0–5 years		3
5–10 years		2
10–15 years		0
15–20 years		3
>20 years		2

**Table 2:** Alpha testing among DTC patients and physicians treating patients with DTC.

	Draft 3: Low-risk DTC patients (*n* = 4)	Draft 3: Advanced disease patients (*n* = 4)	Draft 4: Physician’s low-risk DTC (*n* = 8)	Draft 4: Physician’s advanced disease (*n* = 2)
Length				
Too long	0	0	0	0
Too short	0	0	0	0
Just right	4	4	8	2
Amount of information				
Too much	0	0	0	0
Too little	2	0	0	0
Just right	2	4	8	2
Information balanced?				
Yes	3	4	8	2
Biased towards thyroid lobectomy	1		0	
Biased towards total thyroidectomy	0		0	
Biased towards systemic therapy		0		0
Biased towards watchful waiting		0		0
DA is comprehensible in general?				
Good	4	4	5	2
Moderate	0	0	3	0
Bad	0	0	0	0
Risks comprehensible?				
Good	4	4	5	1
Moderate	0	0	3	1
Bad	0	0	0	0
Readability				
Good	4	3	8	2
Moderate	0	1	0	0
Bad	0	0	0	0
DA reliable?				
Yes	3	4	8	2
No	0	0	0	0
Missing	1	0	0	0
Confusing items?				
Yes	0	0	2	0
No	4	4	6	2
Missing items?				
Yes	0	0	0	0
No	4	4	8	2
Navigation through DA?				
Good	4	2	8	2
Moderate	0	2	0	0
Bad	0	0	0	0
Values clarification exercise helpful?				
Yes	4	4	8	2
No	0	0	0	0
DA helpful in decision making?				
Yes	4	4	8	2
No	0	0	0	0
Recommend use of DA?				
Yes	4	4	8	2
No				

As suggested by Coulter (21), physicians were invited to participate in alpha testing as well. Therefore, the fourth draft was sent to an expert panel of 10 physicians (two endocrinologists, six surgeons, and two oncologists) from academic and nonacademic Dutch hospitals, all specializing in treating patients with DTC. None of them were involved in the initial development of the PtDA. They were asked to assess the PtDA for usability, acceptability of the content and format, practicality of use in the clinical pathways, and their perceived efficacy. They were further asked whether they would be willing to hand out this PtDA to their patients and at what moment in time. Their suggestions were incorporated to create the final fifth version (**Figure 1**), which was deemed acceptable for clinical use. The number of interviews was, again, determined by data saturation (**Figure 1**; **Table 1**). In addition to the quantitative evaluation, the quality of the fourth draft was tested against the IPDAS criteria.

### Beta testing

The beta testing, or “ real-world testing, “ was organized during the ongoing randomized controlled COMBO trial (RCT, NCT03905369). The first 20 participants (**Figure 1**; **Table 3**) in the intervention group who received the PtDA and who were not involved in the design phase were asked to evaluate the feasibility of the PtDA with a questionnaire containing questions on usefulness, length, amount of information, comprehensibility, and reliability (**Table 4**). The quality of the PtDAs was tested using the IPDAS criteria.

**Table 3:** Demographic characteristics of patients who participated in the beta testing.

	Patients (*n* = 20)
**Mean age in years (range)**	52.5 (25–73)
**Sex (*N*, %female)**	14
Level of education	
High school or less	4
Vocational education	12
University	3
Unknown	1
Type of treatment	
Surgery	15
Systemic therapy	5

**Table 4:** Beta testing during the RCT among DTC patients.

	Patients (n=20)
DA used before the conversation with your physician	
Yes	18
No	2
Partly	0
Why not or partly used?	
Too difficult	0
Too much information	0
No time to read the DA	1
No need for a DA	1
	**Patients (n=18)**
How much time (minutes) did you spend on the DA?	45
Values clarification exercise helpful?	
Yes	15
No	3
Better knowledge after reading the DA?	
Yes	15
No	3
Timing	
Too early	0
Too late	6
Just right	12
Length	
Too long	1
Too short	1
Just right	16
Amount of information	
Too much	0
Too little	2
Just right	16
Information balanced?	
Yes	16
No	2
DA is comprehensible in general?	
Good	16
Moderate	2
Bad	0
Readability	
Too easy	1
Too difficult	0
Just right	17
DA reliable	
Yes	18
No	0
Information in a logical order?	
Yes	17
No	1
Grade (0-10)	
0	0
1	0
2	0
3	0
4	0
5	0
6	2
7	6
8	5
9	4
10	1
Recommend use of DA?	
Yes	18
No	0

## Results

### Development process

#### Content and format

**Figure 3:**
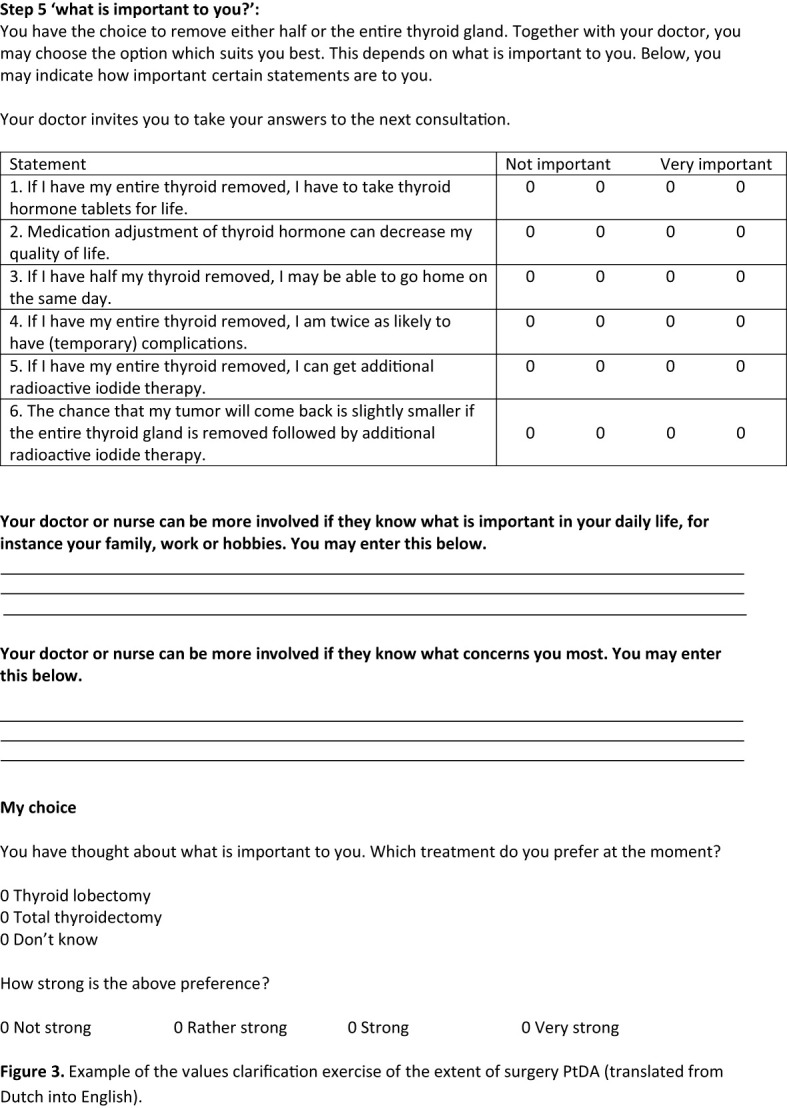
Example of the values clarification exercise of the extent of surgery PtDA (translated from Dutch into English).

Both PtDAs were divided into a general introduction and six steps: general information about DTC; treatment options; comparison of the treatment options; important items; values clarification exercise; and saving the information. The general introduction indicated for whom the PtDA was applicable and contained an explanation of how to use the PtDA. The first step gave general information about DTC. In step 2, the treatment options with the main risks, benefits, and oncological outcomes are presented. In step 3, patients could compare the treatment options. In step 4, patients were asked to answer factual knowledge questions to check for comprehension. Step 5 contained a values clarification exercise with five statements. These were based on patients’ values extracted from the focus group interviews (**Figure 3**) (23). Patients could indicate the importance of each statement on a four-point Likert importance scale ranging from not important to very important. The next page contained two empty boxes. Box 1 asked patients to tell what matters in their lives in general, and box 2 asked for their concerns regarding treatment. They were also asked to indicate which option they preferred and the strength of this preference. In the last step, patients were stimulated to save their answers using email or printing options and to bring these answers along and discuss them with their physicians in the next consultation.

#### Patients and physicians’ expert panel

The second draft of the extent of surgery PtDA was revised by four patients and three physicians. One patient and all three physicians suggested developing two separate PtDAs for “thyroid lobectomy or total thyroidectomy” and “no additional surgery or totalizing thyroidectomy” because of the different treatment options (**Figure 2**). Percentages of complications and a note on life-long adjustment difficulties of substitution with thyroid hormones were added.

The second draft of the TKI PtDA was revised by four patients and five physicians. One patient and all five physicians suggested developing two separate PtDAs, one for sorafenib and one for lenvatinib, the two TKIs that are currently approved for use and reimbursed in the Netherlands, because of different outcome percentages and adverse events (**Figure 2**). More detailed information about the effect of TKIs, adverse events, and information on the palliative character of TKIs were added, as were progression-free survival rates. Both patients and physicians indicated that the general information about DTC was not appropriate for patients with metastatic disease because these patients were already familiar with thyroid cancer, so this information was deleted. In the end, four separate PtDAs for the extent of the surgery decision and the TKI decision were developed (**Figure 2**). Based on this information, the third draft was developed in the form of web-based PtDAs.

### Results of alpha & beta testing and revision

#### Alpha testing 1: patients

The third draft was alpha-tested by four patients treated with surgery and four with advanced disease. Their baseline characteristics are presented in **Table 1**. All patients were satisfied with the content, format, and layout. Length and the amount of information were assessed “just right” by all participants. The information was predominantly judged as balanced and comprehensible, and participants preferred 100-person diagrams for risk communication (31). All participants found the PtDAs useful for decision-making if they had to choose between the treatment options. Participants also suggested some minor changes. To improve the relevance of the extent of surgery, they suggested including more general information about low-risk DTC. To increase the usability of the TKI PtDAs, it was suggested to clarify the navigation through the PtDAs. Lastly, to increase readability, one participant suggested changing the colors of the 100-person diagrams. Other results from the questionnaire are shown in **Table 2**.

#### Alpha testing 2: physicians

The fourth draft was evaluated by 10 physicians (two endocrinologists, six surgeons, and two oncologists) from academic and nonacademic hospitals, all of whom specialized in treating patients with DTC. Their baseline characteristics are presented in **Table 1**. Overall, they were satisfied with the content, format, and layout of the PtDAs. Length and the amount of information were assessed “just right” by all physicians.

For the extent of surgery PtDAs, physicians suggested adjusting the surgery time for thyroid lobectomy as compared to total thyroidectomy because the duration of surgery is not the same for both procedures. There was a discussion about the presented recurrence rates (RR). For the extent of surgery PtDA, physicians suggested reducing the RR for total thyroidectomy from 7% to 4%, based on the following literature regarding the addition of RAI: The mean percentage of RR after the addition of RAI is 4% (32–35). A recent review by Verburg et al. (26) showed that literature published in the last decade offers data that support adjuvant postoperative RAI in DTC patients. Recently, Leboulleux et al. (36) showed in an RCT that the RAI ablation did not result in a significant oncological benefit in patients with low-risk tumors smaller than 2 cm and therefore could possibly be omitted in these cases. The latter represents only a part of the patients targeted by this PtDA, and omitting the RAI ablation for low-risk patients who underwent total thyroidectomy is currently not routinely applied in the Netherlands. All the abovementioned suggestions were admitted in the PtDAs. For the TKI PtDAs, there were no specific suggestions.

Other results from the questionnaire are shown in **Table 2**. All physicians mentioned that they would recommend the use of the PtDAs. **Figure 3** shows the values clarification exercise of the final version of the “thyroid lobectomy or total thyroidectomy” PtDA.

#### Beta testing

The final version was beta- tested during the RCT of the COMBO study. All participants in the intervention group were asked to fill out a questionnaire about the feasibility of the PtDA. A total of 20 participants were already included in this intervention group. Their baseline characteristics are presented in **Table 3**. Two participants did not use the PtDA. The other 18 participants were satisfied with the content, format, and layout of the PtDAs (**Table 4**). Length and the amount of information were assessed “just right” by 89% of participants; the median grade was 8 out of 10, and all participants recommended using the PtDA. Almost all suggested handing over the PtDA as early as possible after the diagnosis, ahead of the decision appointment at home, and also to include more details about their current daily life. They also suggested including the option of active surveillance.

#### IPDAS criteria

The IPDAS collaboration checklist was used to estimate the quality of the PtDAs (20). Of the 64 items on the checklist, 55 quality criteria were applicable to our PtDAs given the scope of the PtDAs. The final version of the PtDAs met 52 out of the 55 applicable IPDAS criteria (95%). Among the 23 criteria for “content,” 21 criteria were met. The two unmet criteria were on listing the option of doing nothing and viewing personalized probabilities based on their own situation. Among the 26 criteria in the “ development process,” 25 were met. The one unmet criterion was the provision of alternative methods to understand the information, such as audio or video options. Lastly, after the beta test, the six criteria for “effectiveness” were all met.

## Discussion

This article describes the systematic development and pilot testing of web-based PtDAs for patients with low-risk (> 1 cm) DTC regarding the extent of primary surgery and for patients with advanced disease regarding starting or delaying the start of TKI treatment. We performed alpha testing with patients and physicians and beta testing with patients. To make the PtDA accessible to every eligible patient, it was written in the A2-B1 language level according to the Common European Framework of for Languages (28). The PtDAs were considered clear, balanced, and helpful for decision-making. The amount of information, length, presentation, and clarity of information received positive feedback. None of the participants indicated that the content was confusing. The criteria for evidence-based PtDA development that have been established by the IPDAS were followed (20). Our PtDAs met 52 of 55 quality criteria for content, development process, and effectiveness as formulated in the IPDAS checklist. Patients acting as reviewers who made a treatment decision in the past indicated they would have preferred to use the present PtDAs, if they had been available at the time of decision-making.

Most, but not all, of the IPDAS criteria were met. For example, listing the option of active surveillance was recommended in the IPDAS criteria. However, we did not include active surveillance in the PtDA for the extent of the surgery decision. At this moment, in national and international guidelines, active surveillance for low-risk DTC patients is not mentioned as a primary treatment option. In recent years, there has been emerging evidence on the safety of active surveillance as an option for the management of micro papillary thyroid carcinomas (mPTCs < 1 cm). More research is necessary before including this option in the PtDA for patients with low-risk tumors larger than 1 cm. When available, these data can be incorporated into a future version of the PtDA. Regarding the criteria to provide information on “viewing probabilities based on their own situation,” we assessed that based on the current knowledge, we could not provide additional information that might better individualize the prediction of the outcomes. For example, although we hypothesize that in general elderly patients have a higher risk of complications or adverse events, length of hospital stay, and mortality (37), we could not find whether and to what extent the risks are higher specifically in elderly DTC patients. A third criterion we did not meet was “the provision of alternative methods to understand the information, such as audio or video options.” Patient participants in our focus group interviews did not prefer audio and/or video options to understand information.

To the best of our knowledge, these are the first documented PtDAs aiming to support DTC patients in these two treatment decisions. Regarding PtDAs in low-risk DTC patients (1–4 cm), there is only one published PtDA focusing on RAI therapy in patients with low-risk papillary thyroid cancer (PTC) (4). This PtDA is limited to the decision to follow or omit RAI treatment after a total thyroidectomy. On the other hand, Brito et al. (38) and Pitt et al. (39, 40) recently developed a treatment choice tool (paper cards) for patients with DTC. These tools included the option of active surveillance, implying that these tools are also useful for informed patients with mPTC. Both tools need further testing before being implemented on a broad scale.

Our PtDAs were designed to facilitate conversations about treatment options for DTC patients in two different treatment decisions. In general, PtDAs have been shown to improve patient knowledge of the health care decision, decrease decisional conflict, and facilitate shared decision-making (18). Patients who use a PtDA are more often satisfied with the choice than those who receive standard counseling (18). However, PtDAs promote conversations between physicians and patients and do not replace the need for a patient–physician consultation. Treatment options still need to be explained to patients to help individualize the trade between harms and benefits according to the patient’s specific situation and clinical situation. PtDAs facilitate a preference-based decision in which patient values and preferences are incorporated (41). By clarifying patients’ values, PtDAs encourage the treatment option that best fits the patient. Therefore, a values clarification exercise was added asking patients to select arguments for and against a treatment option. Feldman-Stewart et al. showed in an RCT in patients with early-stage prostate cancer that values clarification exercises led to better preparation for decision-making and to less regret at the > 1-year follow-up (42).

## Strengths and limitations

A strength of our study is the structured development process of the PtDAs, which systematically uses the input of patients, physicians, and patient advocates. Involving patients in all stages of development yielded important insights. Furthermore, our statements in the values clarification exercise were based on patients’ values extracted from the focus group interviews (23). Another strength is that we developed four very specific PtDAs for each of the decision steps to support decision-making for the full decision trajectory of DTC patients.

A limitation in the development process of the PtDAs is the reliability of evidence regarding the information presented about the clinical outcomes of the different options. This limitation is inherent to the lack of prospective trials on head-to-head comparisons of the treatment options, which can provide the highest level of evidence. Nonetheless, shared-decision making is appropriate for situations where there is insufficient evidence that one option is superior to another, which is also the case for the decisions for which the PtDA has been developed in the present study. Moreover, the information provided in the PtDAs is based on the best available evidence. First, regarding the extent of surgery, only retrospective trials are available, showing no differences in oncological outcomes in terms of RR and OS (6). Second, for the use of TKIs, only two RCTs are available (24, 25). The outcome in terms of OS has not been published yet. Additionally, in daily practice, the effect of TKIs can be different, as the treatment regimens used in practice may differ from regimens used in the RCTs, particularly because of *ad hoc* individualized dose adjustments in patients who are deemed more prone to toxicity or who develop AEs. Furthermore, information desired in rural locations or other countries may differ, potentially making the PtDAs less applicable in settings where less accurate diagnostic tools or less experienced physicians are available. As DTC research continues, new studies may require updating of outcome rates and treatment modalities. Moreover, a prospective randomized controlled trial is ongoing (NCT03905369) investigating the effect and implementation of the PtDAs.

## Conclusion

Novel evidence-based PtDAs were created for patients with DTC. These PtDAs were positively evaluated to support patients and physicians in shared decision-making by patients having undergone the treatments, patient advocates, and physicians. The PtDAs address an important need for DTC patients and aim to increase patient knowledge and guide patients toward an informed decision (23). The PtDAs will be made publicly available after the large prospective trial has been completed.

## Data availability statement

The raw data supporting the conclusions of this article will be made available by the authors, without undue reservation.

## Ethics statement

Ethical review and approval was not required for the study on human participants in accordance with the local legislation and institutional requirements. Written informed consent from the patients/participants or patients/participants’ legal guardian/next of kin was not required to participate in this study in accordance with the national legislation and the institutional requirements.

## Author contributions

AK: conceptualization (equal), writing —original draft preparation (lead), and writing —review and editing (lead). RH: conceptualization (equal) and writing —review and editing (equal). PO: writing —review and editing (equal). RN-M: conceptualization (equal) and writing —review and editing (equal). PS: conceptualization (equal) and writing —review and editing (equal). COMBO study group: conceptualization (supporting). All authors have read and agreed to the published version of the manuscript.

## Group members of COMBO study group

Marieke Snel; m.snel@lumc.nl, Noortje van der Kleij-Corssmit; e.p.m.vanderkleij-corssmit@lumc.nl, Johannes Bonenkamp; han.bonenkamp@radboudumc.nl, Koen Dreijerink; k.dreijerink@amsterdamumc.nl, Evelien van Dam, Grard Nieuwenhuijzen; grardnieuwenhuijzen@catharinaziekenhuis.nl, Mariel Keemers; m.keemers@cwz.nl, Lieke Welling; l.welling@lumc.nl, Iris van der Ploeg; i.vd.ploeg@nki.nl, Sanne Engelen; sanne.engelen@mumc.nl, Danielle Dercks; d.dercks@schildklier.nl, Frans Geenen; fgeenen@xs4all.nl.
